# *In vivo* evolution to hypermucoviscosity and ceftazidime/avibactam resistance in a liver abscess caused by *Klebsiella pneumoniae* sequence type 512

**DOI:** 10.1128/msphere.00423-24

**Published:** 2024-08-22

**Authors:** Valerio Capitani, Gabriele Arcari, Cecilia Ambrosi, Daniela Scribano, Mariateresa Ceparano, Riccardo Polani, Alice De Francesco, Giammarco Raponi, Giancarlo Ceccarelli, Paolo Villari, Anna Teresa Palamara, Carolina Marzuillo, Alessandra Carattoli

**Affiliations:** 1 Department of Public Health and Infectious Diseases, Sapienza University of Rome, Rome, Italy; 2 Department of Molecular Medicine, Sapienza University of Rome, Rome, Italy; 3 Department of Promotion of Human Sciences and Quality of Life, San Raffaele University, Rome, Italy; 4 Laboratory of Microbiology of Chronic-Neurodegenerative Diseases, IRCCS San Raffaele Roma, Rome, Italy; 5 Clinical Microbiology Unit, University Hospital, Policlinico Umberto I, Rome, Italy; 6 Department of Infectious Diseases, Istituto Superiore di Sanità, Rome, Italy; 7 Department of Public Health and Infectious Diseases, Sapienza University of Rome, Laboratory affiliated to Institute Pasteur Italia-Cenci Bolognetti Foundation, Rome, Italy; University of Napoli Federico II, Naples, Italy

**Keywords:** *wzc*, capsule, serum susceptibility, ST512, KPC-31, antibiotic resistance, hmv, hypervirulence, string test, *rmpA *negative, mucoviscous, hypermucoid

## Abstract

Carbapenemase-producing *Klebsiella pneumoniae* represents a major public health issue globally. Isolates with resistance to the newest drugs, like ceftazidime/avibactam (CZA), are increasingly reported. In this study, we analyzed the evolution of KPC-3-producing sequence type (ST) 512 *K*. *pneumoniae* strains isolated at three different times (hospitalization days 45, 56, and 78) from the same patient, two of which were observed in a pericholecystic liver abscess. The three *K. pneumoniae* isolates (295Kp, 304Kp, and hmv-318Kp) from the same patient were subjected to antimicrobial susceptibility testing, whole-genome sequencing, sedimentation assay, biofilm measurement, serum resistance assay, macrophage phagocytosis, and adhesion assays. KPC-producing isolate hmv-318Kp exhibited carbapenem susceptibility, hypermucoviscous (hmv) colony phenotype and CZA resistance. Virulence markers of hypervirulent *Klebsiella* were absent. Two non-synonymous mutations were identified in the hmv-318Kp genome comparing with isogenic strains: a single-nucleotide polymorphism (SNP) occurred in the pKpQIL plasmid, changing *bla*_KPC-3_ in the *bla*_KPC-31_ gene variant, conferring CZA resistance; and a second SNP occurred in the *wzc* gene of the capsular biosynthesis cluster, encoding a tyrosine kinase, resulting in the F557S Wzc protein mutation. The *Klebsiella pneumoniae* strain exhibiting an hmv phenotype (hmv-Kp) phenotype has been previously associated with amino acid substitutions occurring in the Wzc tyrosin kinase protein. We observed *in vivo* evolution of the ST512 strain to CZA resistance and acquisition of hypermucoviscosity. The pathogenetic role of the detected Wzc substitution is not fully elucidated, but other Wzc mutations were previously reported in hmv *K. pneumoniae*. Wzc mutants may be more frequent than expected and an underreported cause of hypermucoviscosity in *K. pneumoniae* clinical isolates.

**IMPORTANCE:**

Here we describe the evolution of KPC-3-producing ST512 *K. pneumoniae* isolated at three different times from the same patient of which the last one, from a biliary abscess, showed CZA resistance by KPC-31 production and manifested hmv colony phenotype. Hypervirulent *Klebsiella pneumoniae* (hv-Kp) isolates are increasingly reported worldwide. Their hypervirulent traits are associated with the presence of rmpA/A2 genes and an hmv. In this study, we identified an hmv-Kp that lacked the rmpA-D cluster but showed an amino acid substitution in the Wzc tyrosin kinase protein, involved in the capsular biosynthesis. This hmv-Kp strain emerged *in vivo* and evolved resistance to ceftazidime/avibactam resistance in a liver abscess of a patient. Our findings suggest that wzc mutations may be underreported, making it challenging to distinguish hv-Kp from “classic” *K. pneumoniae* with an hmv phenotype.

## INTRODUCTION

The *Klebsiella pneumoniae* clonal group (CG) 258 is endemic in several countries and often associated with the production of the *Klebsiella pneumoniae* carbapenemase. In Italy, most the *K. pneumoniae* strains can be assigned to the CG258 (47.4%) lineage, with sequence type (ST) 512 representing the most common clone (1). The KPC class A serine-carbapenemase confers resistance to most beta-lactams, including carbapenems (2). In 2016, the European Medicines Agency authorized the clinical use of ceftazidime/avibactam (CZA), a combination of a third-generation cephalosporin (ceftazidime) with a new-generation non-beta-lactam, beta-lactamase inhibitor (avibactam), to treat serine-carbapenemase-producing *Enterobacterales* (3).

Despite the great activity against KPC, CZA resistance is an emerging issue and is mostly due to the production of KPC variants, with increased ceftazidimase activity and reduced affinity for the avibactam inhibitor (4, 5).

Recent studies have identified a *Klebsiella pneumoniae* strain exhibiting an hmv phenotype (hmv-Kp) (6–8) which is unrelated to the so-called “hypervirulent” pathotype (hvKp) (9). Hypervirulent strains typically feature a virulence plasmid that includes the *rmpA-D* and *iucABCD-iutA* clusters, which contributes to an enhanced virulence phenotype (10). Unlike hvKp, hmv-Kp lacks the *rmpA-D* genes associated with capsular cluster upregulation and the other virulence determinants (11).The genetic determinants and pathogenic potential of hmv-Kp remain largely unclear (12).

In most of the studies, the hmv-Kp phenotype has been associated with amino acid substitutions occurring in the Wzc tyrosin kinase protein, which is one of the major factors responsible for capsule export and polymerization (6, 7, 13, 14). It has been predicted that the autophosphorylation process plays a central role in the Wzc function (15).

The expression of virulence traits like the accessory genes responsible for the hypermucoviscous phenotype in ST512 isolates is anecdotal (16), and no ST512 genome in the Institut Pasteur database (https://bigsdb.pasteur.fr/klebsiella/) carries the associated genes (i.e., *rmpA-D*).

Here we describe an ST512 *K. pneumoniae* strain showing CZA resistance by KPC-31 production and manifested hypermucoviscous (hmv) colony phenotype by Wzc mutation.

## RESULTS

### Case report and phenotypic description of the *Klebsiella pneumoniae* isolates

In January 2022, a patient was transferred to the coronavirus disease 2019 intensive care unit (ICU) of the Policlinico Umberto I (PUI) in Rome, Italy, from another hospital. On day 35 of hospitalization, the patient tested negative for severe acute respiratory syndrome coronavirus 2 and was transferred to the general ICU of the PUI.

On day 45, after 10 days in the general ICU, during a routine gut colonization screening, the patient tested positive for carbapenem-resistant KPC-producing *K. pneumoniae* (isolate 295Kp). On day 49, the patient developed a bloodstream infection caused by a carbapenem-resistant KPC-producing *K. pneumoniae* (isolate not available), concurrent with a clinical and radiological picture of acute complicated cholecystitis with pericholecystitis, as shown by an abdominal CT scan. Empiric antibiotic treatment with fosfomycin, tigecycline, and CZA was started. An ultrasound check on day 56 revealed the presence of a pericholecystic liver abscess, and the patient consequently underwent an emergency cholecystostomy. The procedure was performed under ultrasound guidance using a transhepatic approach. Under fluoroscopic guidance, intrahepatic abscess near the gallbladder bed was drained. The gallbladder was punctured, and an additional cholecystostomy drainage catheter was inserted, with its distal end positioned in the lumen of the gallbladder. The drained material was sent for culture, and the infection was found to be monomicrobial. Carbapenem-resistant KPC-producing *K. pneumoniae* was isolated (isolate 304Kp). Clinically, the patient responded to the antibiotic treatment with fosfomycin, tigecycline, and CZA, resulting in fever resolution and progressive clinical improvement. Twenty-two days later (day 78), a carbapenem-susceptible, CZA-resistant KPC-producing *K. pneumoniae* was isolated from biliary drainage; it showed hypermucoviscous colony phenotype (isolate hmv-318Kp). The treatment with fosfomycin, tigecycline, and CZA was continued until day 88. Given the clinical improvement despite the microbiological isolation, the patient was discharged from the ICU to the transplant surgery ward. On day 90, considering the patient’s asymptomatic condition, the drainage was removed after imaging confirmation of the resolution of the abscess and cholecystitis. Throughout the 90 days of the patient’s hospitalization, the three *K. pneumoniae* isolates (295Kp, 304Kp, and hmv-318Kp), sampled from different sources or exhibiting diverse phenotypes, were subjected to comprehensive analysis for deeper genomics and functional characterization (Table 1; Tables S1 and S2).

**TABLE 1:** Characteristics of the *Klebsiella pneumoniae* sequence type 512 strains analyzed in this study

Isolate	Source^*a*^	Day^*b*^	Minimal inhibitory concentration (mg/L)^*c*^		KPC	hmv^*d*^	Wzc
			CZA	MEM			
295Kp	RS	45	0.5	>8	KPC-3	Negative	WT
304Kp	BA	56	0.19	>8	KPC-3	Negative	WT
hmv-318Kp	BA	78	12	2	KPC-31	Positive	F557S

^*a*^ Abbreviations of the strain samples: RS, rectal swab; BA, biliary abscess.

^*b*^ Day of isolation of the strain considering day 1 as the first day of hospitalization.

^*c*^ CZA, ceftazidime/avibactam; MEM, meropenem.

^*d*^ Hypermucoviscosity was measured by string test.

### Comparative genomic analyses among the three isolates

By genomic analyses, the three isolates were assigned to ST512 by *K. pneumoniae* multilocus sequence typing (MLST) (11) and were negative for the presence of the *rmpA-D* genes, lacking all genetic determinants associated with the typical virulence plasmid causing the hypervirulent *Klebsiella pneumoniae* (hv-Kp) phenotype. The only siderophore present in all the three genomes is yersiniabactin, and by Kleborate tool, the resulting virulence score is 1 (17).

A total of nine single-nucleotide polymorphism (SNPs) were detected on the chromosome sequence, comparing the initial isolate 295Kp from the rectal swab and the first 304Kp isolate from the liver abscess: two caused synonymous substitutions; five caused missense mutations in various metabolic proteins; and one caused a frameshift mutation in a galactosidase protein (Table S1). A recombination event was identified in 304Kp pKpQIL with respect to 295Kp pKpQIL, involving the region of the transfer locus. One SNP (synonymous mutation) was identified on the 304Kp pKpQIL, in the *traN* gene.

All differences identified between 295Kp and 304Kp were also confirmed in hmv-318Kp. Only three additional SNPs were found, comparing 304Kp and the hmv-318Kp the strains isolated from the liver abscess at 22 days of distance. One SNP was identified in the pKpQIL located *bla*_KPC-3_ gene, defining the *bla*_KPC-31_ variant conferring CZA resistance, another one was identified in the chromosomal gene encoding the tyrosine kinase Wzc defining the F557S variant.

A third SNP was identified in the intergenic region adjacent to the repressor of biofilm formation gene *bssS*. This mutation involved neither the –35 nor the −10 promoter sequences of the gene. No differences in the number or position of insertion sequences were observed.

### Analyses of the hypermucoviscous phenotype

**Fig 1:**
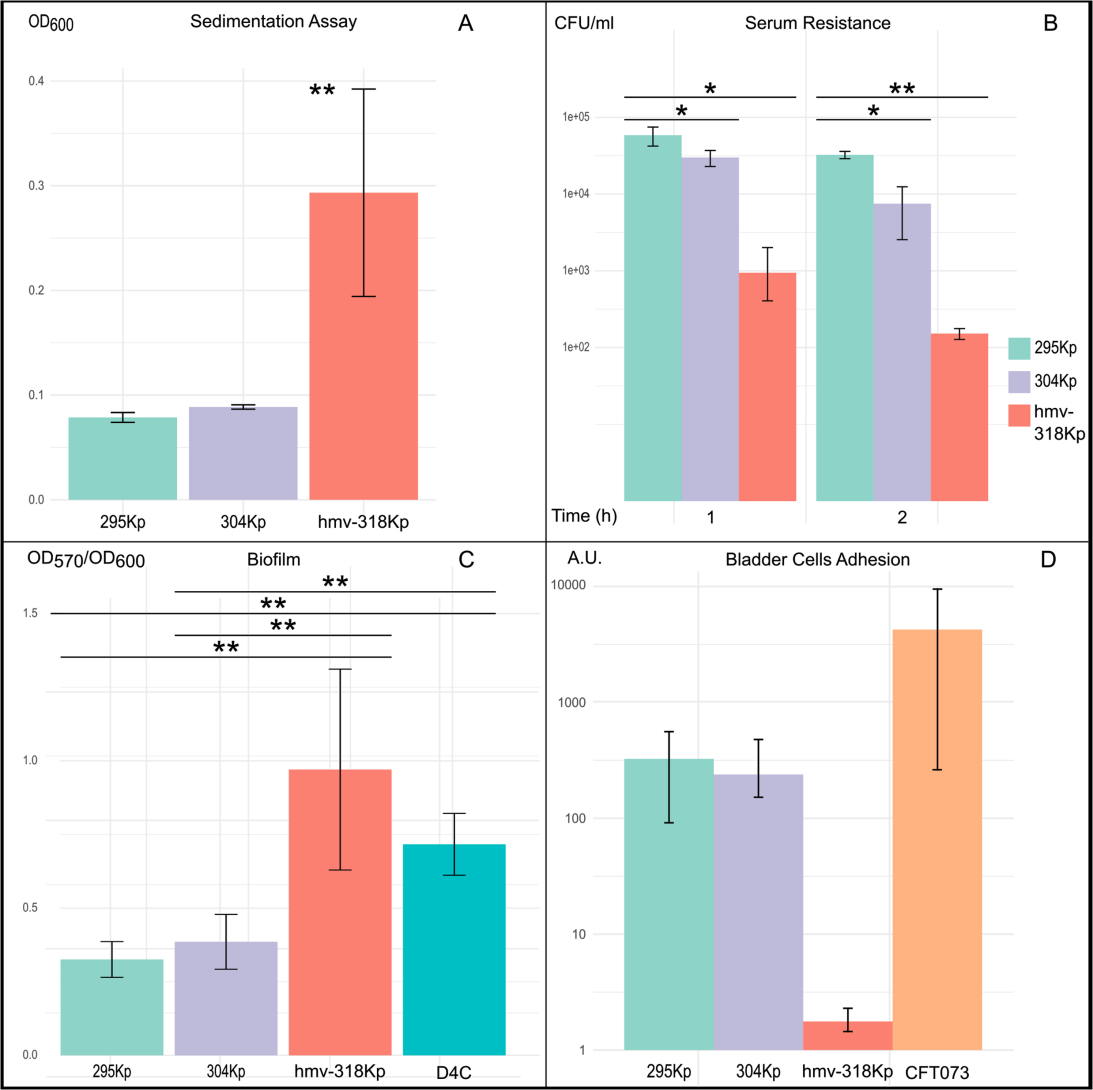
Phenotypic characterization of *Klebsiella pneumoniae* sequence type 512 analyzed in this study. (**A**) Quantitative sedimentation assay. Isolates, grown overnight in brain heart infusion, were normalized to OD_600_ = 1.0 centrifuged at 1,000 × *g* for 5 min, and the OD_600_ of the upper 900 µL was measured. Data are shown as means + SDs (*n* = 6). (**B**) Serum resistance assay. Tolerance to human sera pool was evaluated by exposing bacteria (5 × 10^5^ CFU/mL) to 100% human serum and incubating at 37°C for 2 h. CFU/mL were enumerated at T0, after 1 and 2 h. Data are shown as means + SDs (*n* = 6). (**C**) Quantitative assessment of biofilm formation. Biofilm formation was measured after 24-h incubation under static conditions and reported as the OD_570_:OD_600_ ratio to normalize the amount of biofilm formed to the total bacterial content. Data are shown as means + SDs (*n* = 48). (**D**) Adhesion assay to human bladder epithelial cells. Confluent HTB-9 cell monolayers were infected with each isolate using a multiplicity of infection of 100. Cell surface-adherent bacteria were enumerated after 3-h incubation. The uropathogenic *Escherichia coli* strain CFT073 was included as a positive control. Data are shown as means + SDs (*n* = 9) and expressed as percentage considering arbitrarily strain 295Kp as the reference. Asterisks above bars represent *P* values evaluated by one-way analysis of variance: *P < 0.05, **P < 0.01.

Out of the three strains under examination, only hmv-318Kp tested positive to the string test (>2.0 cm in length). The hmv-318Kp strain showed higher sedimentation assay values. Sedimentation assay mean values determined for isolates 295Kp and 304Kp were very similar to each other, being OD_600_ = 0.077 and OD_600_ = 0.089, respectively, while the mean value for the hmv-318Kp isolate was almost four times higher, being OD_600_ = 0,293 (Fig. 1A).

The hmv-318Kp strain showed increased susceptibility to human serum, with a mortality rate two logarithms higher after 1 h of exposure in human serum, further increasing after 2 h (Fig. 1B).

Isolates 295Kp and 304Kp exhibited a modest ability to form biofilm, while hmv-318Kp displayed a significantly greater ability to form biofilm, when compared to the D4C *Escherichia coli* strain, used as a positive control (Fig. 1C).

**Fig 2:**
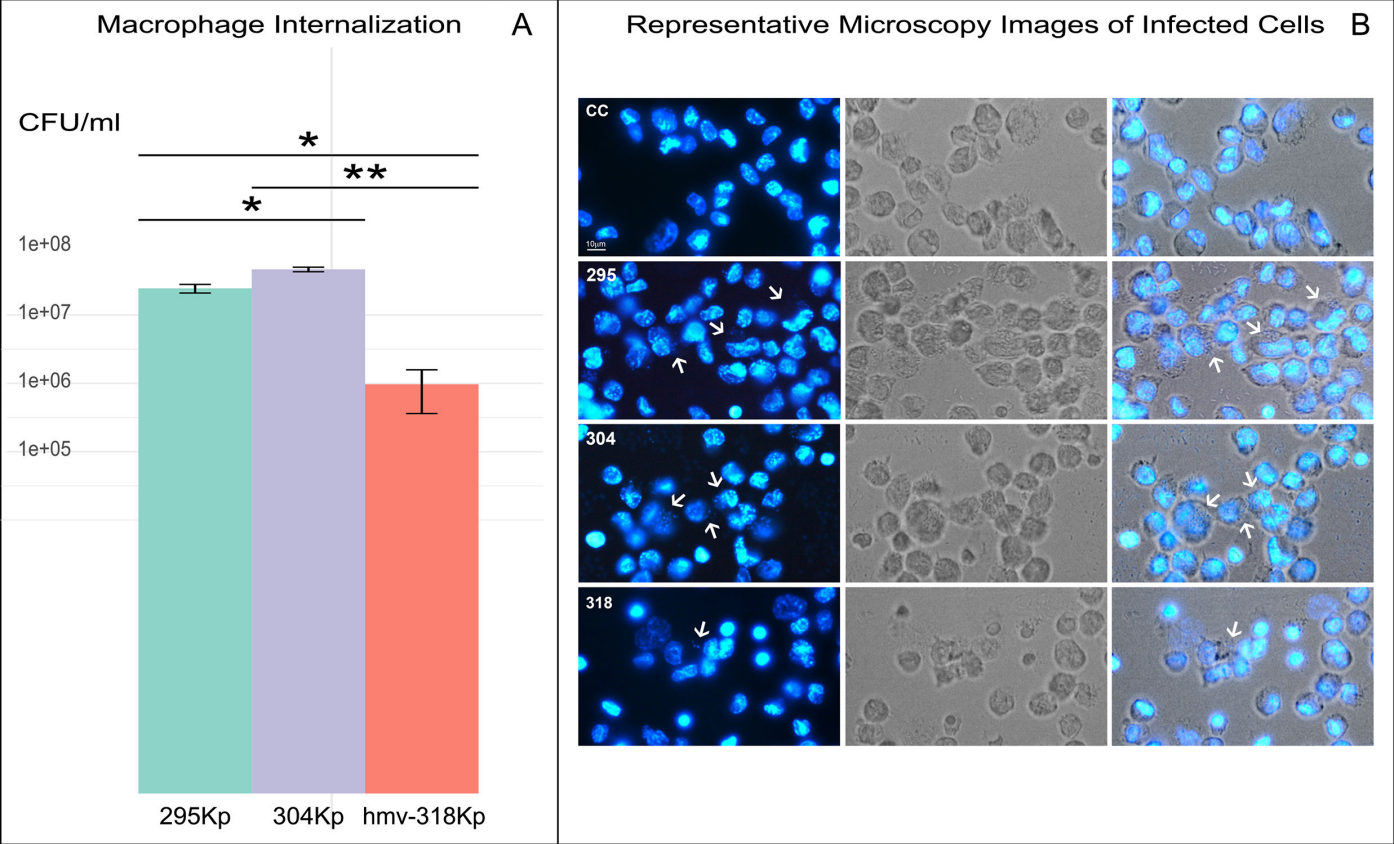
Evasion from phagocytosis by human macrophages of *Klebsiella pneumoniae* sequence type 512 strains isolated in this study. (**A**) Macrophage internalization assay. Confluent THP-1 cell monolayers were infected with each isolate using a multiplicity of infection of 100. Two hours after infection, chloramphenicol at 60 µg/mL was added to the medium to kill extracellular bacteria. Internalized bacteria were enumerated after an additional 1 h of incubation. Data are shown as means + SDs (*n* = 9). Asterisks above bars represent *P* values evaluated by one-way analysis of variance: *P < 0.05, **P < 0.01. (**B**) Representative microscopy images of infected cells. THP-1 cell monolayers infected in parallel were acquired in bright and fluorescence fields 4′,6-diamidino-2-phenylindole (DAPI). Scale bar = 10 µm.

Testing the ability to adhere to bladder epithelial cells, hmv-318Kp exhibited lower adherence than 295Kp and 304Kp and was almost completely removable from the epithelium after 3 h. However, all three *Klebsiella* isolates exhibited lower adhesion ability compared to the adhesive strain of *E. coli* CFT073, which served as a positive control (Fig. 1D). In the human macrophage evasion test, 295Kp and 304Kp strains were almost completely internalized by macrophages (Fig. 2), while hmv-318Kp exhibited a 10-fold reduction of internalization, suggesting that the hypemucoviscous strain has improved ability to evade phagocytosis by macrophages.

## DISCUSSION

In this article, we describe the *in vivo* evolution of colonizing and infectious isolates of the ST512 *K. pneumoniae* strain isolated from a single patient. In particular, comparing the two isolates from the liver abscess, we identified only two SNPs located in non-coding regions, one in *bla*_KPC-3_, becoming the *bla*_KPC-31_ gene variant able to confer CZA resistance (4), and the second in the *wzc* gene, encoding the protein-tyrosine kinase involved in exopolysaccharide and capsular polysaccharide biosynthesis and transport (13). The Wzc F557S variant is the most likely cause of the hmv-colony phenotype observed in the isolate hmv-318Kp.

In *K. pneumoniae*, the hmv phenotype is canonically associated with the *rmpA*-D locus (for regulator of mucoid phenotype A). RmpA plays the role of enhancer of the capsular polysaccharide biosynthesis, by cooperation with RcsB transacting factor in modulating the regulatory activity. RmpD is responsible for the hv-Kp phenotype interacting with the protein Wzc (18). However, the hmv phenotype is not necessary linked to capsule overexpression (19). Wzc mutations have been previously described to be responsible for the hmv phenotype without other hypervirulence-related determinants (14, 19). Mutations in the *wzc* gene have been previously associated with hmv phenotypes in ST258 in the USA (7) and in ST307 and ST15 in China (6) but occurred in different aminoacidic residues. Actually no *wzc* nucleotide sequence in the National Center for Biotechnology Information databank is the same as the one reported here (Wzc F557S). All these previous examples were sporadic isolates from different strain collections, and the functional role in pathogenesis for *wzc*-mutants still is not clearly defined.

Our patient case allows to open some hypotheses about the properties of the emerged mutant and how it survived. It is possible that in the liver abscess, the isolate was not accessible to sufficient doses of antimicrobials that, along the time, selected the conversion of *bla*_KPC-3_ in the CZA resistant variant *bla*_KPC-31_. Furthermore, we measured for the hmv phenotype a better evasion of phagocytosis by macrophages that potentially confer an advantage in the abscess context. This analysis confirmed previous reports describing a better ability of *wzc* mutants to evade this type of host defense. The hmv-318Kp has a lower ability to survive in serum when compared with the other non-hmv isogenic isolates. This strain lacked the capacity to adhere to bladder epithelial cells. This cell type was tested to verify the known effects of the Wzc mutations as described by Ernst and colleagues (7). Therefore, phenotypic characteristics of the hmv-318Kp conform to previous observations (8) but undermine the hypothesis of functional Wzc mutations as a factor favoring the bloodstream spread of *K. pneumoniae* (7). Strangely, we observed that hmv-318Kp has a greater ability to form biofilm. These data partially contrast with previous research, despite employing similar measurement methods (7).

A limitation of this article is that we were unable to identify the specific cause of the enhanced biofilm formation ability of hmv-318Kp. This may be due to the mutation identified upstream of the *bssS* gene, although it does not involve the −10 and −35 promoter sequences.

Our description also opens the hypothesis that *wzc* mutations are probably underreported in “classic” *Klebsiella* showing the hypemucoviscous phenotype. Increasing numbers of reports are describing hypemucoviscosity in isolates lacking the *rmpA-D* genes, but the determinants associated with this phenotype were not identified (20–25). This implies that effectively recognizing hv-Kp from classic *K. pneumoniae* with hmv phenotype represents an even more complex challenge than expected.

## MATERIALS AND METHODS

### Isolation, identification, and collection of *K. pneumoniae* strains

Bacteria were isolated during routine diagnostic procedures from the intensive care unit of the Policlinico Umberto I University Hospital.

Species identification was performed by matrix-assisted laser desorption ionization–time of flight mass spectrometry system (Bruker Daltonik GmbH, Bremen, Germany), and antimicrobial susceptibility testing was performed by the Vitek2 system (bioMérieux, Marcy-l'Étoile, France). Fosfomycin MIC was confirmed using the gold-standard agar dilution method (Liofilchem, Roseto degli Abruzzi, Italy), and CZA MIC was confirmed using gradient strips (Liofilchem).

All “ESKAPE” pathogens (26) isolates sampled from the PUI ICU are stored for epidemiological purposes. To collect the isolates, those are grown on brain heart infusion (BHI) agar plates (Oxoid Limited, Basingstoke Hampshire, United Kingdom), incubated overnight at 37°C, resuspended in glycerol 10% (wt/wt) Luria-Bertani broth, and stored at −80°C.

Three prototypical isolates (listed in order of sampling: 295Kp from rectal swab, 304Kp and hmv-318Kp from biliary abscess) displaying phenotypic differences were collected and analyzed (Table 1).

### String test

*K. pneumoniae* strains were grown at 37°C overnight on 5% sheep blood agar and MacConkey agar plates. A standard 10-µL bacteriological loop was used to stretch a single colony, and the formation of a continuous filament >5 mm long was considered as a positive string test (27).

### Sedimentation assay

Sedimentation assay was performed in triplicate on the three isolates under examination. The sedimentation assay was adapted from previously described protocols (28). Isolates were grown overnight in BHI at 37°C in agitation at 100 rpm. The overnight cultures were centrifuged at 16,000 × *g* for 15 min, the obtained pellet was resuspended at OD_600_ = 1.0 in a final volume of 1 mL phosphate-buffered saline (PBS). Samples were centrifuged at 1,000 × *g* for 5 min, and the OD_600_ of the upper 900 µL was measured.

### Serum resistance

Whole blood was collected from two healthy volunteer donors. After 30 min at room temperature (RT), the clot was removed by centrifuging at 1,500 × *g* for 10 min in a refrigerated centrifuge. The resulting supernatants, designated as sera, were pooled together. Overnight bacterial cultures were normalized, measuring OD_600_, and 1 mL of each culture was centrifuged at 13,000 × *g* for 5 min, washed twice with an equal volume of PBS. Then, 50 µL of each culture corresponding to 5 × 10^5^ CFU/mL for each strain was added to 1 mL of pooled sera, as described by Guo et al. (29). CFUs per milliliter were enumerated at T0, after 1 and 2 h. Three independent experiments were repeated, each carried out in duplicate.

### Infection assays

Cultured HTB-9 (ATCC-LGC, Milan, Italy) human bladder epithelial cell line and THP-1 (ATCC TIB-202) human monocytes (differentiated to macrophages with 50 nM of 12-O-tetra-decanoylphorbol-13-acetate for 48 h) were used for infection experiments, as previously described (30). Cell monolayers were cultured in Dulbecco's modified Eagle medium (DMEM) and RPMI 1640, respectively, at 37°C under 5% CO_2_ atmosphere until 90% confluency and were infected using a multiplicity of infection of 100. To synchronize the infection, plates were centrifuged at 540 × *g* for 10 min. Bladder cells were infected for 3 h, while macrophages were incubated with bacteria for 2 h followed by 1 h in the presence of chloramphenicol at 60 µg/ml. Cell monolayers were washed five times with PBS to remove unbound/extracellular bacteria and subsequently treated with 1 mL of 0.1% Triton X-100 for 5 min. Following lysis, bacteria were quantified by plating out 10-fold dilutions of the bacterial suspensions. Quantifications were performed in triplicate on 3 different days, and the mean results were expressed as percentage, considering arbitrarily strain 295Kp as the reference. The well-characterized uropathogenic *E. coli* strain CFT073 was included as a positive control for adhesion assays (31).

### Biofilm measurement

Biofilm formation was measured using the microtiter plate assay, as previously described (32, 33). Briefly, overnight bacterial cultures were normalized to 1 × 10^9^ cells/mL, and 20 µL was added to 180 µL of LB. Microplates were incubated for 24 h under static conditions. After washing, biofilms were fixed with methanol and stained with crystal violet (1%) for 15 min at RT. Crystal violet was solubilized with ethanol, and absorbance was measured at 570 nm. Results are reported as the OD_570_/OD_600_ ratio to normalize the amount of biofilm formed to the total bacterial content. Four independent experiments, 12 wells per strain (*n* = 48), were performed.

### Whole-genome sequencing and bioinformatic analyses

Whole-genome sequencing was carried out, performing both short- and long-read technologies on every isolate. Specifically, short-read Illumina sequencing (Illumina Inc., San Diego, CA, USA) was performed on genomic DNA purified using the Isolate II genomic DNA Extraction Kit (Bioline, Cincinnati, USA), while long-read Oxford Nanopore Technologies (ONT) sequencing (ONT, Oxford, UK) was performed on high-molecular-weight genomic DNA extracted using the Monarch HMW DNA Extraction Kit for Tissue (New England Biolabs, Massachusetts, USA). Unicycler v.0.4.8.0 (34) was used with standard polishing parameters and a normal bridging mode to combine the Illumina performed by SPAdes (35) and the ONT assembly obtained by Flye (36), and its results were refined by the Bandage tool (37).

The resulting assemblies were typed by PlasmidFinder (38) to assess plasmid content and by Kleborate (11) to detect virulence-associated and anti-microbial esistance (AMR) genes.

SNPs among the three genomes were analyzed using the Snippy tool (https://github.com/tseemann/snippy), comparing Unicycler v.0.4.8.0 final sequences and gbk files annotated by Bakta (39). Promoter prediction was performed using Softberry Prediction of Bacterial Promoters (http://www.softberry.com). The insertion sequences are identified with ISfinder (40).Recombinant regions were predicted using Gubbins (41), and the annotated genomes were visualized with Proksee (42). When possible, bioinformatic analyses were carried out via the public Galaxy.eu server (https://usegalaxy.eu) (43).

## Data Availability

Biosamples, Sequence Read Archive, and whole-genome sequencing have been released for the three genomes under study in BioProject (PRJNA1100498). The complete genomes and plasmid content of isolates 304Kp and hmv-318Kp have been released under accession numbers JBCPSL000000000 and JBCPSK000000000, respectively.

## SUPPLEMENTAL MATERIAL

Table S1 and S2 msphere.00423-24-s0001.pdf Genomic and phenotypic differences among the three strains analyzed in this study.

ASM does not own the copyrights to Supplemental Material that may be linked to, or accessed through, an article. The authors have granted ASM a non-exclusive, world-wide license to publish the Supplemental Material files. Please contact the corresponding author directly for reuse.
