# Supplementary material for: In vivo evolution to hypermucoviscosity and ceftazidime/avibactam resistance in a liver abscess caused by Klebsiella pneumoniae sequence type 512
Source: mSphere. 2024 Aug 22;9(9):e00423-24. doi: 10.1128/msphere.00423-24 (PMC11423586; doi:10.1128/msphere.00423-24)
Supplement: Table S1 and S2 — Genomic and phenotypic differences among the three strains analyzed in this study. [file msphere.00423-24-s0001.pdf]

Table S1: Genomic differences identified in 295Kp, 304Kp and hmv-318Kp strains analysed in this study

**Chromosomal SNPs between**			**295Kp**	**304Kp**									
CHROM	POS	TYPE	REF	ALT	EVIDENCE	FTYPE	STRAND	NT_POS	AA_POS	EFFECT	LOCUS_TA(	GENE	PRODUCT
contig_1	1871472	snp	G	T	T:20 G:0	CDS	+	346/1434	116/477	stop_gained (	ELPMDN_0	bglB	Beta-glucosidase/beta- galactosidase
contig_1	2298056	snp	A	G	G:20 A:0	intergenic region							
contig_1	2909947	snp	T	C	C:20 T:0	CDS	-	590/1089	197/362	missense_va	ELPMDN_1	frmA	Zn-dependent alcohol/formaldehyde dehydrogenase
contig_1	2911568	snp	T	C	C:20 T:0	CDS	+	784/1845	262/614	synonymous_	ELPMDN_1	mdoD	Glucans biosynthesis protein D
contig_1	3080614	snp	T	C	C:20 T:0	CDS	+	223/618	75/205	synonymous_	ELPMDN_14905		Histidine phosphatase
contig_1	3432109	snp	G	A	A:20 G:0	CDS	-	56/951	19/316	missense_va	ELPMDN_1	qor	NADPH:Zn-dependent oxidoreductase
contig_1	4355380	snp	A	T	T:20 A:0	CDS	-	1211/1257	404/418	missense_va	ELPMDN_2	mscS	Small-conductance mechanosensitive channel
contig_1	4356272	snp	T	A	A:20 T:0	CDS	-	319/1257	107/418	missense_va	ELPMDN_2	mscS	Small-conductance mechanosensitive channel
contig_1	4668481	snp	A	T	T:20 A:0	CDS	+	227/540	76/179	missense_va	ELPMDN_22750		DUF3251 domain-containing protein

Plasmid_2 SNPs	POS	TYPE	REF	ALT	EVIDENCE	FTYPE	STRAND	NT_POS	AA_POS	EFFECT	LOCUS_TA(	GENE	PRODUCT
contig_2	93051	snp	A	G	G:20 A:0	CDS	-	1801/2007	601/668	synonymous_	ELPMDN_2	traN	type-F conjugative transfer system protein TraN

Recombination on	POS_START	POS_END
contig_2	48972	56043

**Chromosomal SNPs between**			**304Kp**	**hmv-318Kp**									
CHROM	POS	TYPE	REF	ALT	EVIDENCE	FTYPE	STRAND	NT_POS	AA_POS	EFFECT	LOCUS_TA(	GENE	PRODUCT
contig_1	2224236	snp	T	C	C:20 T:0	CDS	+	1670/2157	557/718	missense_va	DJHHHM_1	wzc	Polysaccharide biosynthesis tyrosine autokinase
contig_1	3808283	snp	C	A	A:20 C:0	intergenic region							

Plasmid_2 SNPs	POS	TYPE	REF	ALT	EVIDENCE	FTYPE	STRAND	NT_POS	AA_POS	EFFECT	LOCUS_TA(	GENE	PRODUCT
contig_2	59077	snp	C	A	A:20 C:0	CDS	-	532/882	178/293	missense_va	DJHHHM_2	blaKPC-3	carbapenem-hydrolyzing class A beta-lactamase KPC-3

Staramr comparison

	**295Kp**	**304Kp**	**hmv-318Kp**
Chromosome			
AMR genes	OqxA	OqxA	OqxA
AMR genes	OqxA	OqxA	OqxA
AMR genes	OqxB	OqxB	OqxB
AMR genes	OqxB	OqxB	OqxB
AMR genes	blaSHV-182	blaSHV-182	blaSHV-182
AMR genes	fosA6	fosA6	fosA6
Plasmid_1	IncFIB(pQil)	IncFIB(pQil)+Inc	IncFIB(pQil)+IncFII(K)
AMR genes	blaKPC-3	blaKPC-3	blaKPC-31
AMR genes	blaOXA-9	blaOXA-9	blaOXA-9
AMR genes	blaTEM-1A	blaTEM-1A	blaTEM-1A
Plasmid_2	Unknown	Unknown	Unknown

Plasmid_3	ColRNAI	ColRNAI	ColRNAI
AMR genes	aac(6')-Ib	aac(6')-Ib	aac(6')-Ib

Table S2: Phenotypic differences identified in 295Kp, 304Kp and hmv-318Kp strains analysed in this study

Measurement	1	2	3	4	5	6	7	8		
Biofilm	OD570/OD600	OD570/OD600	OD570/OD	OD570/OD	OD570/OD	OD570/OD	OD570/OD	OD570/OD	Average valu	Standard deviation
295Kp	0,304	0,358	0,325	0,313	0,492	0,394	0,461	0,356	0,375	0,069
304Kp	0,677	0,357	0,423	0,473	0,387	0,475	0,385	0,351	0,441	0,107
hmv-318Kp	1,475	0,758	0,712	1,278	1,796	0,939	1,172	0,775	1,113	0,389
D4C	0,985	0,862	0,986	0,694	0,713	0,713	0,787	0,842	0,823	0,118

	Antibiotic	Amoxicillin/Ac cl	Piperacillin/tazobact	Cefoxitin	Cefotaxime	Ceftazidim	Cefepime	Ertapenem	Meropenen	Amikacin	Gentamicir	Ciprofloxac	Tigecyclin	Fosfomycir	Colistin	Trimethopri	Ceftazidime/Avibactam
295Kp	MIC	>16	>64	>32	>32	>32	>16	>4	>8	16	>8	>2	2	64*	<=0,5	<=20	0.5*
295Kp		R	R	R	R	R	R	R	R	I	R	R	I	S	S	S	S
304Kp	MIC	>16	>64	>32	>32	>32	>16	>4	>8	16	>8	>2	2	128*	<=0,5	<=20	0.19*
304Kp		R	R	R	R	R	R	R	R	I	R	R	I	R/R	S	S	S
hmv-318Kp	MIC	>16	16	>32	>32	>32	>16	>4	2	16	>8	>2	4	64*	<=0,5	<=20	12*
hmv-318Kp		R	I	R	R	R	R	R	S	I	R	R	R	R/S	S	S	R

* Ceftazidime/avibactam MIC is obtained by gradint strips; Fosfomicyn MIC is obteined by agar plate microdiluitions

MIC interpretation is based on EUCAST 2023 parameters
